# U-Shaped Development: An Old but Unsolved Problem

**DOI:** 10.3389/fpsyg.2013.00301

**Published:** 2013-05-27

**Authors:** Franz Pauls, Thorsten Macha, Franz Petermann

**Affiliations:** ^1^Center of Clinical Psychology and Rehabilitation, University of Bremen, Bremen, Germany

**Keywords:** U-shaped functions, developmental psychology, diagnostic decision-making, test development, non-monotonic development

## Abstract

Even today the investigation of U-shaped functions in human development is of considerable importance for different domains of Developmental Psychology. More and more scientific researchers focus their efforts on the challenge to describe and explain the phenomenon by identifying those skills and abilities being affected. The impact of U-shaped functions on diagnostic decision-making and on therapeutic treatment programs highlights the importance of understanding the nature of non-monotonic development. The present article therefore addresses the relevant questions of how U-shaped functions are defined in theory, in which developmental domains such non-monotonic growth curves are suggested to occur, and which implications there are for future methodology and diagnostic practice. Finally, it is recommended to clearly identify those interactions between proximal and distal subcomponents which are expected to contribute to a U-shaped development.

The explanation of intraindividual changes in behavior, as well as the identification of interindividual variations in such changes across the life span are regarded as the overall task of developmental science (Baltes et al., 1977). A developmental function represents one of the most valuable tools for describing those variations. Wohlwill (1973) defined this function as “… the form or mode of the relationship between the chronological age of the individual and the changes observed to occur in his responses on some specified dimensions of behavior over the course of his development to maturity….”

As is often the case with special phenomena in human development, U-shaped functions highlight some fundamental problems in need of explanation. In contrast to a typically suggested monotonic improvement of skills and abilities with age, U-shaped functions restrict the number of possible explanatory approaches. This is because each of those functions has to conclusively refer to domain-specific stages of development. It has to be proved in individual cases why skills initially emerge early in life and why they disappear with age to re-emerge at a time yet to be determined. The examination of U-shaped functions in child development seems to be of increasing interest these days because the function’s mere appearance inevitably stimulates the generation of appropriate explanations.

Therefore, the major aim of the current article is to raise awareness for the phenomenon of U-shaped development itself and to offer the opportunity of getting a deeper insight into the nature of U-shaped functions. Although it is beyond the scope of this article to discuss when and to what extent U-shaped functions are valid, however, we are initially operating on the assumption that they exist. By reviewing previous attempts to explain U-shaped functions, we first intend to clarify what reasons there might be for the appearance of U-shaped functions in development and in which developmental domains such functions have already been empirically supported to occur. On this basis, we then address the question of which implications there could be for test development and diagnostic practice. Finally, some theoretically and practically oriented recommendations are given in order to improve future curve modeling and diagnostic decision-making in psychotherapy.

## Why are U-Shaped Functions Occurring in Human Development?

As Siegler (2004) emphasized, the question of how U-shaped functions make a substantial contribution to Developmental Psychology is closely connected to the question of what they can tell us about the underlying processes. For this purpose, Siegler presented three main ways in which declining performances within U-shaped functions might be produced.

First of all, he stated that the acquisition of a more systematic processing strategy in childhood might result in a decremented performance on specific tasks. In that context, a U-shaped course of development is characterized by initial correct performances based on a lack of knowledge, followed by consistently incorrect performances as a result of incomplete knowledge. These are later superseded by correct performances based on advanced knowledge and experience (Siegler, 1981, 1983). Namy et al. (2004) found that 18 months old infants are able to connect certain gestures with corresponding referents regardless of their iconicity. But as children begin to perceive that gestures are iconically related to their referents, they become less skillful in connecting iconically unrelated gestures and referents. As they grow older, children then become more skillful with increasing experience.

U-shaped functions could also be explained by the adoption of novel processing strategies. This adoption might initially result in a cognitive overload and in temporary losses of processing efficiency (Werker et al., 2002). Bjorklund et al. (1997) had come to the conclusion that executing novel strategies often imposes high demands on cognitive resources. This easily decimates the pool of those resources which remain for the actual task. Once novel processing strategies have been completely adopted, they lead to a more efficient cognitive processing.

Finally, Siegler suggested the unequal rates of change in certain monotonically improving processes to be responsible for a U-shaped course of development. When Pinker and Prince (1988) analyzed the U-shaped development of producing irregular past tense forms in English, their findings supported the concept of parallel distributed processing in language acquisition. At the outset, linguistic experience seems to strengthen a rule-based representation of regular past tense forms. At this stage, lexical representations of irregular past tense forms are more likely to be outpaced due to the predominance of regular forms in language (Siegler, 2004). With increasing certainty, rule-based processing enables to use correct irregular past tenses again (but see Rumelhart and McClelland, 1987).

To illustrate possible mechanisms which could underlie the dynamic nature of a U-shaped development, a brief overview of previous empirical findings is given in the following section.

## Are there Empirically Derived Links to Developmental Psychology?

At present developmental psychologists appear to agree that the U-shaped development of certain human abilities represents a transition from a prior developmental episode to a higher-level one. A temporary regression to the effect that certain abilities are present early in life and disappear to re-emerge at a later age, is suggested to be deeply rooted in the instability of maturation. Given that some already acquired abilities seem to wither at a certain stage of life, although being fully developed before, one could conclude that human development is characterized by a very fluctuant and fragile nature.

Concerning the development of motor skills, Thelen et al. (1982) reported a U-shaped development of step-like movements at birth. In later studies, Thelen and Ulrich were again able to show that the disappearance of stepping from 2 to 12 months of age is for the most part caused by the increase in body weight. To demonstrate a causal relationship, Thelen and Ulrich reduced the need for muscle strength by placing infants in water, and step-like movements were recovered in children who previously revealed no stepping response. These findings thus indicated the adaptation and interaction of unequally developing entities (Thelen and Ulrich, 1991). The linearly increasing weight of the head and limbs coupled with the more slowly increasing strength of the leg and neck muscles could be seen as one reason for the disappearance and reappearance of step-like movements. For a long time, scientific researchers believed that typing speed of professional typists should slow down due to a continuous decrease in their ability to react with increasing age. Prevailing perspectives rather indicate a U-shaped function than a continuous diminution in typing speed. Although aged typists clearly display slower reaction times as compared with younger colleagues, older typists tend to slightly outperform younger typists on tasks measuring typing speed (Uttal and Perlmutter, 1989). In their study, Uttal and Perlmutter (1989) proved that older typists increased their letter span as keystroke speed declined. A wealth of experience and an extensive expertise in typing could both make up for deficits in reaction. Regarding language development, Ervin and Miller (1963) suggested that early stages of speech comprehension are most notably marked by the reintegration of different subsystems following a period of disorganization. Before children are able to produce the correct past tense of irregular verbs, almost all of them go through a period where they initially tend to overregularize (Bybee and Slobin, 1982). The acquisition of mentally represented irregular verbs enables children to systematically use the correct past tenses again (Marcus et al., 1992; Plunkett and Juola, 1999; but see Rumelhart and McClelland, 1987).

Further empirical support for the existence of U-shaped functions is to be found for perception-related abilities (Werker et al., 2002; Kuhl et al., 2006; Sebastián-Gallés and Bosch, 2009), motor coordination (Butterworth and Morisette, 1996), eye-hand coordination (von Hofsten, 1982, 1984; Hay et al., 1991), and further verbal skills (Jackson and Cottrell, 1997; Swingley, 2009). Even scientific investigations dealing with the development of creativity support the idea of U-shaped patterns in development (Davis, 1997).

Since previous research has already substantiated a consistent appearance of such a phenomenon, in any case, we believe that the existence of U-shaped functions cannot *a priori* be denied at all. In our opinion, U-shaped functions should always be interpreted in due consideration of domain-specific aspects of the ability in question. Having access to appropriate diagnostic tools is not least an important requirement for such investigations. The foundation for a valid identification, description, and explanation of U-shaped functions in development should already be laid in the early stages of test construction. Therefore, the question of what problems might arise from those functions concerning test development and test construction is addressed in the following section.

## Which Implications are there for Test Development and Test Construction?

It is often assumed that test items of a given diagnostic instrument differentiate between individuals on a single difficulty continuum. All items within the same scale then have to measure exactly the same ability but should feature different degrees of difficulty. Oppenheim (1981) already suggested that certain abilities are only developing in dependence of further changing distal and proximal variables. Such abilities would then be insufficiently assessed by unidimensional tests. It should be also considered that a test’s content and its required cognitive demands could be qualitatively changing with age. A diagnostic instrument that was originally designed to include simple discrimination tasks for infants might turn out to demand complex learning processes when applied to older children. Empirical evidence for various effects of confounded variables on development can be found for sensory perception (Lewkowicz and Turkewitz, 1980) and motor coordination (Uttal and Perlmutter, 1989; Thelen and Ulrich, 1991). As long as levels of confounded abilities are allocated to a unidimensional scale, a dimensional shift in development easily results in a misconception about the true stage of development. Development profiles which are purely based on quantitative and unidimensional measures often fail to disclose the complexity of intraindividual maturation processes. Finally, analyzing quantitative data and cross-checking it with qualitative information should help to verify the existence of domain-specific U-shaped functions. Otherwise the interpretation of such non-monotonic developmental functions might be quite ambiguous, particularly if other multicausally related variables are not taken into consideration. A well-established method to prove homogeneity of a diagnostic instrument is to verify its unidimensionality by conducting a factor analysis whenever quantitative measures are to be assessed. Given that a single factor is extracted as required for a unidimensional scale, the portion of explained variance then indicates a certain level of item homogeneity. To prove a test’s dimensional identity across different age groups, it should be cross-validated by comparing it to external measures. These could be instruments either measuring the same trait and being applicable to comparable age groups (convergent validity) or measuring a similar but distinct trait (divergent validity).

Test construction in Developmental Psychology mostly follows the rules and axioms of Classical Test Theory (CTT; see, Allen and Yen, 2002, for a detailed description). It is usually suggested that difficulty parameters of the given test items are conducted so as to monotonically increase with age (Petermann and Macha, 2005, 2008). Just as developmental testing has to be carried out in an age-based manner, corresponding difficulty parameters have to be calculated for each single age group under examination. It means that these parameters finally describe a point-by-point course of change with increasing age, thus being expected to represent a certain developmental function. Depending upon which limits of age-related intervals have been selected for the investigation, U-shaped patterns might indeed not be identified due to the missing data for those age groups that were not examined. Test construction therefore should be at least …

(a) based on a sufficiently large sample size that
(b) provides a sufficiently high concentration of values among the entire age range.

**Figure 1:**
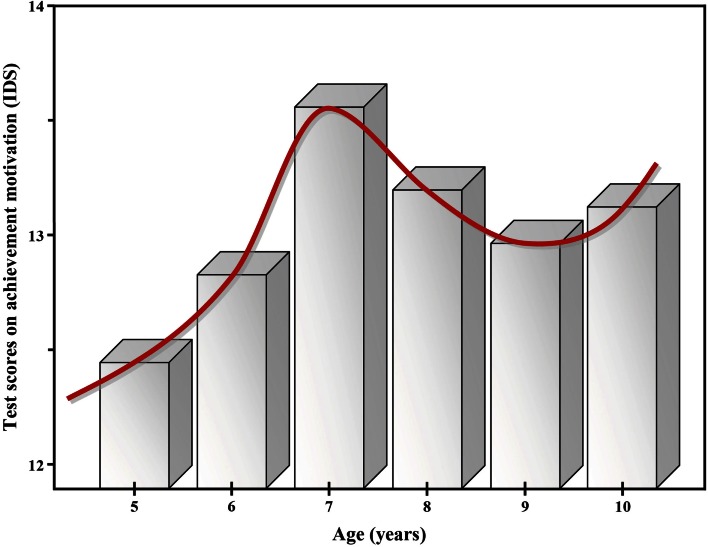
**Mean test scores on the IDS subscale “achievement motivation” changing with age: an example of a true U-shaped development between 7 and 10 years of age?**.

In practice, however, test items that do not reveal parameter characteristics in accordance with a monotonic improvement in development are either totally excluded from the final test version or simply attributed to nothing but methodological deficiencies. Developmental psychologists publishing U-shaped functions such as illustrated for achievement motivation (IDS; Grob et al., 2009) are rather the exception than the rule (Figure 1). Nevertheless, it can be assumed that non-monotonic courses in development are more likely to be observed than is indicated by the currently available literature.

## What are the Consequences of U-Shaped Functions for Developmental Diagnostics?

A developmental test which fails to provide valid and reliable data based on repeated measurements reduces the general quality of diagnostic investigations. Furthermore, it could frustrate any purposeful attempt to detect atypical functions in development. Even small deviations within a development profile which might be attributable to nothing but psychometric inadequacies of the test itself could be over-interpreted at the content level.

**Figure 2:**
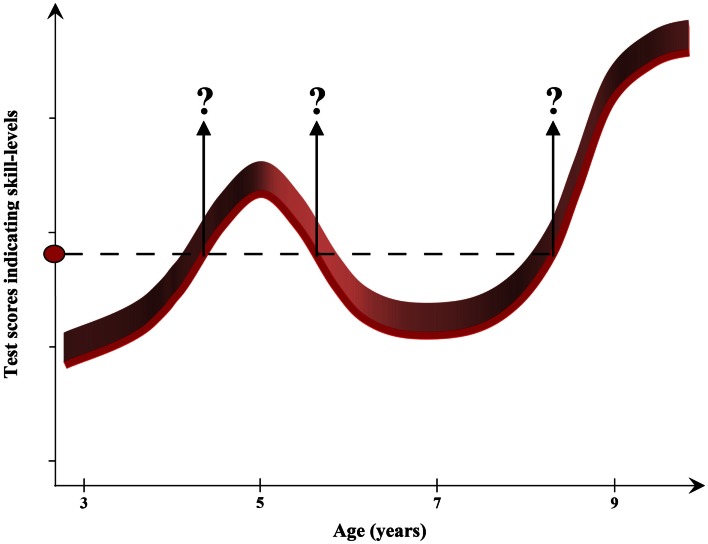
**Example for a U-shaped development: the marked skill-level is achieved at three different ages**.

Interpreting individual test performances and assigning correct levels of development might be a considerable challenge for diagnosticians whenever different stages of development are suggested to follow a U-shaped function. Two individuals of different ages could then be allocated to the same performance level. Figure 2 illustrates the characteristic developmental function for the accuracy of aiming in direction (Hay et al., 1991). The discontinuous line defines a certain performance level which was achieved by children of 4.5, 5.5, and 8.5 years of age. If specific performance levels are estimated purely on the basis of test results, then it is virtually impossible to determine a child’s definitive position on the developmental continuum without ambiguity. It could then be quite difficult for diagnosticians to comprehend whether and why the same level of functioning is shown in two individuals of different ages.

Qualitatively related and confounded variables which contribute to specific stages of development may easily lead to flawed diagnostic decisions whenever they are quantified by test results. Interpreting such test results by merely evaluating how many deviations the achieved scores are above or below the mean (e.g., expressed in standard scores like *Z*-scores, *T*-scores, *IQ*-scores, or standard nine), might be insufficient and nonsensical depending on the diagnostic context.

Whenever U-shaped functions are expected to characterize the development of diagnostically relevant proficiencies, it is advisable to split the overall test performance into subcomponents and to assess them separately. For example, the accuracy of aiming in direction might include monotonically improving perceptual, cognitive, and motor components. These subcomponents should be assessed separately from each other to be at least able to capture specific developmental core elements. The general sensitivity and specificity of a diagnostic instrument could also be increased for the benefit of an accurate identification of clinically conspicuous symptoms.

Despite the fact that many empirical findings support the occurrence of U-shaped functions in different developmental domains, there remains a critical stance toward these results as well. It still has to be clarified whether empirically obtained patterns do really reflect true courses of developmental change, or whether a U-shaped function represents nothing but a methodological artifact. Developmental functions which may vary depending on whether children were tested once a year, once a month, or every day (Siegler, 2006; Adolph et al., 2008), highlight the need for measurements to be improved by proper experimental designs and testing conditions. It has to be noted that a variety of different diagnostic techniques are required on the part of developmental psychologists depending on whether sucklings, infants, or preschool age children are to be examined (Macha et al., 2005).

## Implications for Future Methodology and Diagnostic Decision-Making

Although numerous U-shaped functions were identified within different fields of Developmental Psychology down to the present day, however, research findings still appear to be fragmentary to some extent. Therefore, future research based on fruitful scientific cooperation is indispensible when it comes to lay the groundwork for gaining new insights into the nature of U-shaped development.

Current methodological innovations have an immediate effect of increasing the priority given to the goal of more accurate descriptions, simply by making them possible. Morse et al. (2011) presented a promising neuro-computational approach to modeling cognitive development. Their model is primarily based on refinements of the associative learning mechanism as part of the Epigenetic Robotics Architecture (ERA). The ERA technique enables and facilitates the predictions for stage-like developmental transitions by data simulation (Morse et al., 2010). The opportunity of modeling non-linear growth curves is of great value to developmental science because several crucial development characteristics can be estimated this way. Generalizable models on the basis of which reliable development-related predictions are to be made at least require two important assumptions about the function characteristics. First, intraindividual changes in development should follow the same general developmental function across different individuals. Second, intraindividual variations in developmental changes should be relatively stable throughout the entire duration of development. Assuming that a U-shaped function might mirror a general developmental process, researchers use different growth models (Grimm et al., 2011) to provide developmental theories with detailed information about within-person and between-person differences in change (McArdle, 1988; Meredith and Tisak, 1990; Bryk and Raudenbush, 1992).

The phenomenon of a U-shaped development plays a decisive role for diagnostics within therapeutic treatment programs. If U-shaped functions can be considered to result from an integration of new processing strategies, then similar developmental patterns ought to be generated by reorganizational processes within a therapy as well. Just consider that a child is often faced with the instruction to quit its old and inadequate behavioral strategies in favor of novel target-oriented ones during a common therapeutic process. Although being psychotherapeutically supported, the same child could initially suffer losses in performance due to cognitive overload. A temporary regression here could result from the treatment itself before the child’s capability improves up to an age-appropriate level again. Such an initiated U-shaped development that would actually support therapeutic effectiveness in the given context, however, might be misinterpreted by therapists that do falsely assume a monotonic improvement. In the most unfavorable case, a highly effective psychotherapeutic treatment could then be discontinued according to a seeming lack of success. It is therefore necessary for future clinical psychologists to take these considerations about U-shaped functions into account when developing new therapeutic treatment programs, evaluating the efficiency of those being currently conducted, or analyzing individual development profiles.

In future, diagnostic procedures should be optimized to identify those interacting subcomponents which are said to contribute to the development of the ability in question. A more systematic analysis of the interactions between those subcomponents would then provide the opportunity to extract helpful information about clinically significant indicators for developmental change.

## Conflict of Interest Statement

The authors declare that the research was conducted in the absence of any commercial or financial relationships that could be construed as a potential conflict of interest.
